# Mycotic pulmonary artery pseudoaneurysm following total arch replacement: a case report

**DOI:** 10.1186/s40792-024-01896-9

**Published:** 2024-05-03

**Authors:** Hiroaki Osada, Hisashi Sakaguchi, Kazuhiro Yamazaki, Kenji Minatoya

**Affiliations:** Department of Cardiovascular Surgery, Graduate School of Medicine, Kyoto University, 54 Shogoin-Kawaharacho, Sakyo-ku, Kyoto, 606–8507 Japan

**Keywords:** Mycotic pseudoaneurysm, Infective endocarditis, Graft infection

## Abstract

**Background:**

Although the true prevalence and incidence are not clearly known, mycotic pulmonary artery aneurysm is a potentially devastating condition that leads to high mortality, over 60% if untreated. Among them, mycotic pulmonary artery pseudoaneurysm, which occurs in relatively central areas, has rarely been reported. We report an extremely rare case of a late complication with a mycotic pulmonary artery pseudoaneurysm, presumably due to infective endocarditis, in a 68-year-old woman 4 months after total arch replacement.

**Case presentation:**

A 68-year-old woman was referred to our department for 2 weeks with fever of unknown origin. She had a history of emergency total arch replacement for an acute type A aortic dissection 4 months earlier and chronic rheumatoid arthritis on monthly subcutaneous tocilizumab treatment for several years. Blood culture was positive for *Enterococcus faecalis*. Transthoracic and transesophageal echocardiography revealed a left ventricular ejection fraction of 58%, severe mitral regurgitation with a 15-mm diameter vegetation on the anterior mitral leaflet, and severe aortic insufficiency with string-like structures. Contrast computed tomography showed a focal saccular outpouching from the right pulmonary artery. On 18F-fluorodeoxyglucose (FDG) positron emission tomography, focal uptake of FDGs was observed along the same lesion of the pulmonary artery and ascending-arch graft. The patient eventually recovered after the surgical intervention of mitral and aortic valve replacement, re-total arch replacement, pulmonary artery repair, application of omental flap, and antibiotics without any evidence of re-infection after 1 year.

**Conclusions:**

We report a successful surgical repair of mycotic pulmonary artery pseudoaneurysm 4 months after total arch replacement for acute type A aortic dissection. This report describes an effective treatment for an extremely rare postoperative condition.

## Background

Although the true prevalence and incidence are not clearly known, mycotic pulmonary artery aneurysm (PAP) is a potentially devastating condition that leads to high mortality, over 60% if untreated [1]. Among them, mycotic PAP, which occurs in relatively central areas, has rarely been reported [2]. We report an extremely rare case of a late complication with mycotic PAP, presumably due to infective endocarditis, in a 68-year-old woman 4 months after total arch replacement (TAR).

## Case presentation

A 68-year-old woman was referred to our department for 2 weeks with fever of unknown origin. She had a history of emergency TAR for an acute type A aortic dissection 4 months earlier and chronic rheumatoid arthritis on monthly subcutaneous tocilizumab treatment for several years. At the initial surgery, the patient presented with paraplegia and urinary tract infection as postoperative complications and was transferred to another facility for rehabilitation.

**Fig. 1:**
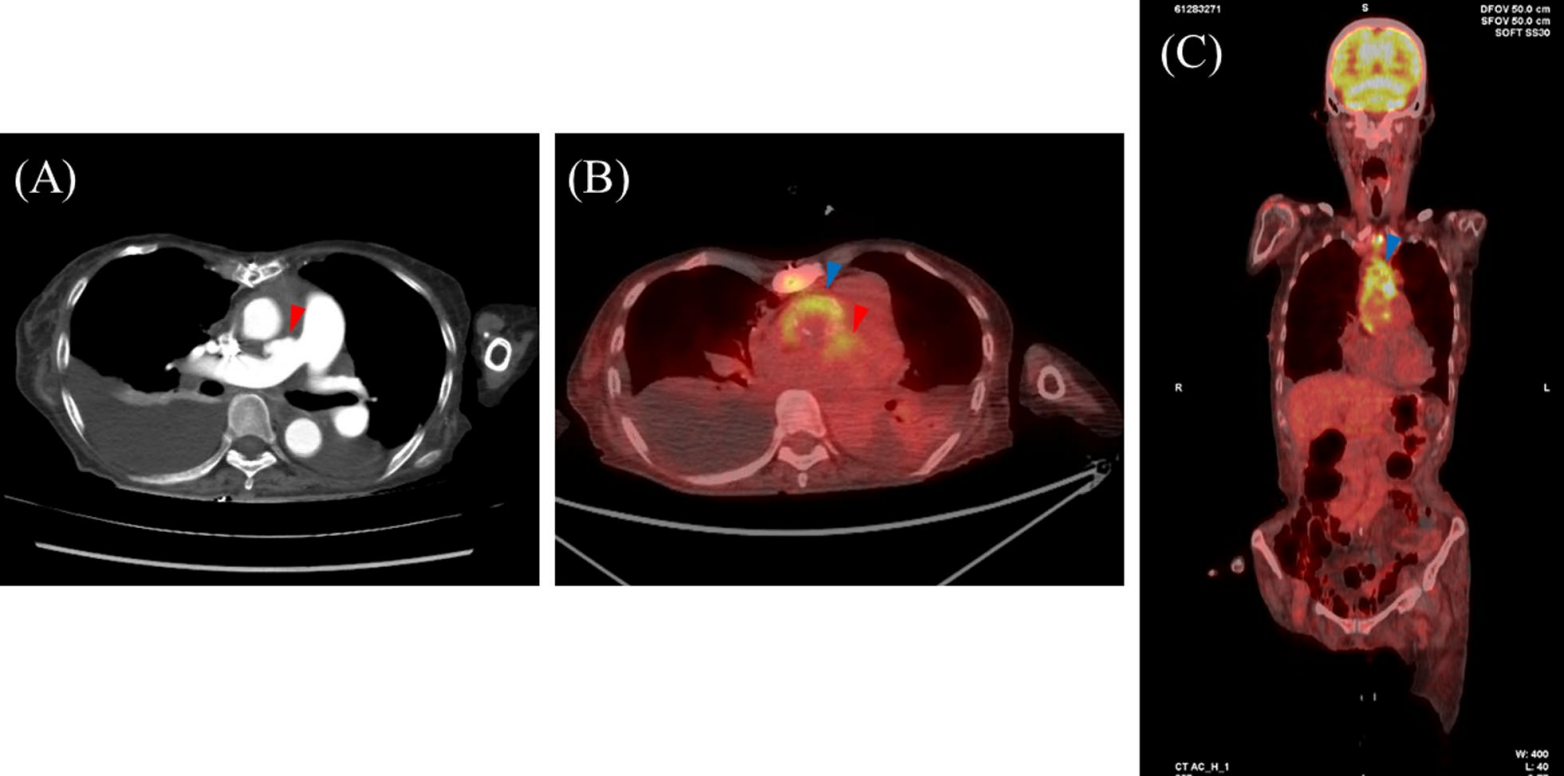
**A** Preoperative contrast CT showed a focal saccular outpouching from the right pulmonary artery (red arrowhead). **B**, **C** Preoperative FDG-PET showed focal uptake of FDGs along the PAP (red arrowhead) and ascending-arch graft (blue arrowhead)

A blood culture was positive for *Enterococcus faecalis*. Transthoracic and transesophageal echocardiography revealed a left ventricular ejection fraction of 58%, severe mitral regurgitation with a 15-mm-diameter vegetation on the anterior mitral leaflet, and severe aortic insufficiency with string-like structures. Contrast computed tomography (CT) revealed a focal saccular outpouching from the right pulmonary artery (Fig. 1A). On ^18^F-fluorodeoxyglucose positron emission tomography (FDG-PET), focal uptake of FDGs was observed along the same lesion of the pulmonary artery and ascending-arch graft (Fig. 1B, C).

Infective endocarditis and ascending-arch graft infection, concomitant with mycotic PAP, were strongly suspected. Because of the size of the vegetation and rapid progressive heart failure, urgent surgery was performed. Before re-sternotomy, the femoral artery and vein were taped through a right inguinal incision in preparation for cannulation. Upon re-sternotomy, the pericardial space was dissected. Because of sudden massive bleeding from the dorsal aspect of the ascending aortic graft, while manual control of the bleeding was attempted, a cardiopulmonary bypass with ascending side branch cannulation and bi-caval venous drainage was hastily initiated, and systemic cooling was started simultaneously.

**Fig. 2:**
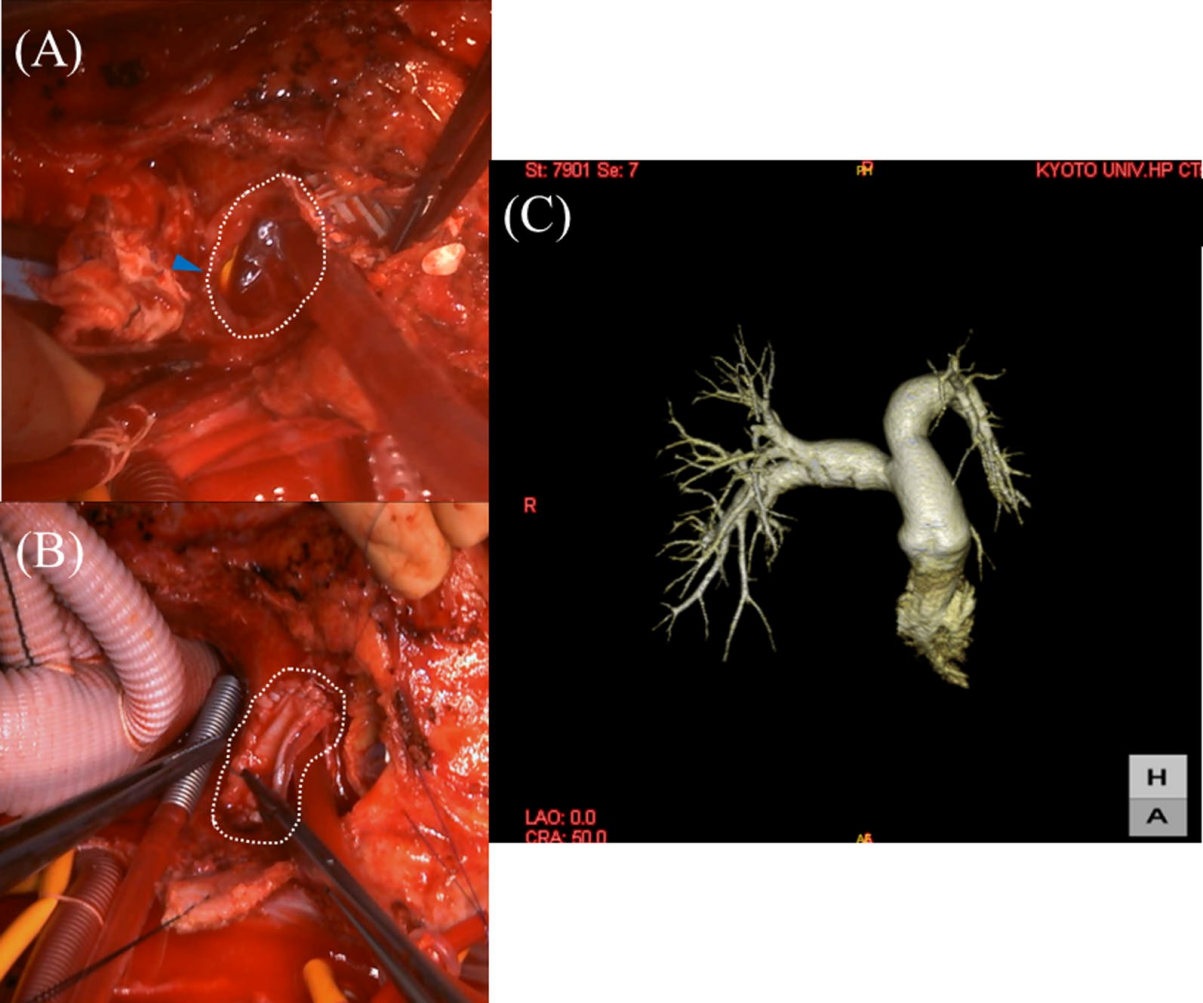
**A** Operative image after aortic graft clamping, removal of ascending aortic graft. A large defect was found ventral to the right pulmonary artery (white dotted line). The blue arrowhead shows the Swan–Ganz catheter in the pulmonary artery. **B** Operative image after PAP repair using a bovine pericardial patch (white dotted line). **C** Postoperative 3D-CT revealed favorable reconstruction of the right pulmonary artery

After cross clamping of the ascending aortic graft, the proximal aortic graft was removed and a large defect was found ventral to the right pulmonary artery (Fig. 2A). In addition, disruptions were noted on the dorsal aspect of the proximal suture line. Observation of the mitral valve revealed large vegetation on the anterior mitral leaflet. Both leaflets were removed and replaced with a 27-mm Epic mitral bioprosthesis (Abbott Medical Japan, Co. Ltd, Tokyo, Japan). The aortic valve was also replaced with a 21-mm Edwards Inspiris RESILIA aortic bioprosthesis (Edwards Lifesciences, Irvine, CA, USA). Re-TAR was then performed with a 22-mm 4-branched rifampicin bonded graft (Gelweave Ante-Flo Gelatin Impregnated Woven Dacron Graft, Sulzer Vascutek, Renfrewshire, Scotland) using antegrade cerebral perfusion. Reconstruction of the right pulmonary artery was performed using a bovine pericardial patch (Fig. 2B). Finally, an omental flap was applied to the anterior mediastinum before sternal closure. The operation time was 668 min and the aortic cross clamp time was 231 min. Culture tests performed intraoperatively detected *Enterococcus faecalis* in the mitral valve, aortic valve, ascending-arch graft, and PAP.

Fever resolved immediately after surgery. Postoperative 3D-CT revealed favorable reconstruction of the right pulmonary artery (Fig. 2C). The patient was discharged after 6 weeks of intravenous double-therapy with antibiotics (CTRX, ABPC/MCIPC), followed by lifelong oral AMPC. Currently, after 1 year, careful monitoring is in progress without any evidence of re-infection.

## Discussion

PAP has been reported to be associated with trauma, vasculitis, and neoplasms, whereas infection-related “mycotic pseudoaneurysm” is caused by tuberculosis and pyogenic bacteria. In many cases, pseudoaneurysm forms in the peripheral pulmonary arteries and is associated with respiratory symptoms such as hemoptysis and shortness of breath [1, 3–5]. Although the true prevalence and incidence are not clearly known, mycotic PAP is a potentially devastating condition that leads to high mortality, over 60% if untreated [1]. Among them, mycotic PAP, which occurs in relatively central areas, has rarely been reported [2].

In this case, at the initial surgery, the patient presented with paraplegia and urinary tract infection as postoperative complications. In addition, the patient was using immunosuppressive medication for rheumatoid arthritis. It is undeniable that these conditions contributed to new infections. Further attention is required for these patients in the future, including when to resume immunosuppressants during the initial postoperative period. On the other hand, examining the mechanism of the present condition, the tricuspid and pulmonary valves were intact, and mild–moderate mitral regurgitation and mild–moderate aortic insufficiency remained on echocardiography before discharge of initial surgery. It is presumed that the mycotic PAP was triggered by infective endocarditis in the left heart system, leading to infection of the ascending-arch graft and weakening of the proximal suture line, which in turn directly infected the right pulmonary artery. During the initial surgery, the dissection did not extend into the sinus of Valsalva, and glue was not used during the proximal reconstruction. No damage or repair was recorded in the right pulmonary artery. Tissue necrosis or injury to the right pulmonary artery is considered unlikely.

Fortunately, the patient eventually recovered after the surgical intervention. Although there may be a wide variety of factors contributing to this condition, urinary tract infection from paraplegia may have been a factor, reminding us of the importance of infection control in the perioperative period. If the presence of a pseudoaneurysm had not been recognized preoperatively, this case could have turned lethal, even intraoperatively, thus reminding us of the importance of preoperative contrast assessment.

## Conclusions

We report a successful surgical repair of mycotic pulmonary artery pseudoaneurysm 4 months after total arch replacement for acute type A aortic dissection. This report describes an effective treatment for an extremely rare postoperative condition.

## Data Availability

The datasets used and/or analyzed in the current study are available from the corresponding author on reasonable request.

## Abbreviations

3D: 3-Dimensional
CT: Computed tomography
FDG-PET: 18F-fluorodeoxyglucose positron emission tomography
PAP: Pulmonary artery pseudoaneurysm
TAR: Total arch replacement

## References

[1] Benhassen LL, Højsgaard A, Allan Terp K, de Paoli FV (2018). Surgical approach to a mycotic aneurysm of the pulmonary artery presenting with hemoptysis—a case report and a review of the literature. Int J Surg Case Rep.

[2] Kalra-Lall A, Donaldson J, Martin C (2019). Brief review: pulmonary artery aneurysms and pseudoaneurysms. Int J Cardiovasc Imaging.

[3] Chen Y, Gilman MD, Humphrey KL, Salazar GM, Sharma A, Muniappan A (2017). Pulmonary artery pseudoaneurysms: clinical features and CT findings. AJR Am J Roentgenol.

[4] Guillaume B, Vendrell A, Stefanovic X, Thony F, Ferretti GR (2017). Acquired pulmonary artery pseudoaneurysms: a pictorial review. Br J Radiol.

[5] Pujitha V, Shaw M, Kumar S, Ramakrishnan S (2022). A rare case of infective endocarditis in association with ventricular septal defect complicated by mycotic pulmonary artery aneurysm. J Card Surg.

